# Analyzing Instagram Food and Nutrition Posts Through a Food Literacy Lens: Content Analysis of Instagram Posts

**DOI:** 10.2196/22272

**Published:** 2025-11-12

**Authors:** Yalinie Kulandaivelu, Jill Hamilton, Ananya Banerjee, Anatoliy Gruzd, Jennifer Stinson

**Affiliations:** 1 University of Toronto Toronto, ON Canada; 2 Hospital for Sick Children Toronto, ON Canada; 3 McGill University Montreal Canada; 4 Toronto Metropolitan University Toronto Canada

**Keywords:** social media, Instagram, food literacy, healthy eating, nutrition, content analysis

## Abstract

**Background:**

Dietary behaviors are directly linked to health and well-being. Food literacy education may improve poor dietary behaviors and thus, health and well-being. Social media is a popular source of food literacy education through content delivered by influencers and experts alike. Characterizing food and nutrition content on social media using a food literacy framework can identify gaps in public food literacy knowledge and opportunities for improving food literacy education.

**Objective:**

The primary objective of this study was to systematically characterize and categorize publicly available food- and nutrition-related Instagram content according to food literacy concepts.

**Methods:**

We conducted a mixed methods study using content analysis. We collected Instagram posts that used hashtags related to the term “healthy eating” via CrowdTangle. We completed our content analysis using Netlytic to categorize posts according to our framework of food literacy and topics of interest. Then, we completed a descriptive qualitative content analysis of a sub-sample of posts from each category.

**Results:**

Our analysis included 100,000 Instagram posts. We categorized the Instagram posts using 19 categories related to food literacy and attitudes to healthy eating. The most frequent categories were (1) information about foods to consume (38,500/100,000, 38.5%), (2) cooking and preparing food (36,007/100,000, 36%), and (3) planning and managing food intake (33,262/100,000, 33.3%). Protein-rich foods, fiber, vegetables, juicing and smoothie diets, and spices were commonly promoted as foods to consume, while selecting organic and fresh foods was encouraged more frequently than canned or frozen foods. Processed and prepared foods were discouraged. Baking was frequently portrayed as a cooking method, as well as quick and easy recipes, and cooking with friends and family. Planning food intake was frequently discussed in relation to weight loss and holidays. Cultural foods were portrayed as healthy foods and with healthier variations, and in the context of holidays and religious observances. Low-cost and affordable foods were portrayed with minimal time requirements, minimal ingredients, and depicted as family-appropriate.

**Conclusions:**

Instagram content frequently portrayed healthy eating as part of a healthy lifestyle and impacting physical health, activity and energy levels, and mood. However, prescriptive information regarding foods to consume was still pervasive. Encouragement to cook together and share recipes together indicates the social aspect of eating and cooking as important to users and may be an important aspect of food literacy guidance and programs in the future. Our descriptive analysis of Instagram content demonstrates several opportunities for supporting and improving food literacy education on social media.

## Introduction

Dietary behaviors are strongly related to health and well-being. Poor diet quality has been directly linked to the development of chronic illnesses, poor mental health, and reduced ability to engage in activities of daily living and physical activity [1-5]. Dietary behaviors are influenced by a complex set of factors, including food security, which is the availability, affordability, and accessibility of foods in the environment; hunger, appetite, and taste; media, cultural and social norms, and family and peers; mood, stress and guilt; attitudes and beliefs about food; and food literacy [6-10]. Dietary behaviors change during the transition to adulthood as young people face the challenges of living independently alongside increasing control over their diets [11]. The dietary behaviors that are established during this period of transition seldom change later in adulthood and are a significant predictor of future health [12,13]. Thus, food literacy education has emerged as an important modifiable component of a multipronged strategy for improving dietary behaviors [14]. Food literacy is defined as “the scaffolding that empowers individuals, households, communities, and nations to protect diet quality through change (eg, in income, location, culture, and life circumstances) and strengthen dietary resilience over time. It is composed of the collection of interrelated knowledge, skills, and behaviors required to plan, manage, select, prepare, and eat food to meet needs and determine intake” [15]. These skills and knowledge are socially shaped and lay outside the control of health care providers [15]. Thus, social media has emerged as a popular source of food literacy education [16].

Adults and adolescents alike are more likely to look for and use nutrition information from social media than other sources [17,18]. Social media is used to actively search for recipes, cooking ideas, and nutrition information [16,19] as well as passively be exposed to food-related content through suggested posts identified by social media algorithms [20]. Social media allows laypersons and experts alike to share food and nutrition information. Laypersons sharing nutrition information on social media may gain more attention from other users and be perceived as credible when posting about health-related issues, even if they are not health professionals [20]. This is due to their shared background and life circumstances with users, along with their ability to communicate health information in a way that is accessible to users [21]. Therefore, social media permits users who actively or passively identify posts and accounts relatable to themselves to engage with food literacy on social media.

When a layperson reaches a large number of followers, they are usually referred to as an “influencer” [22]: from Microinfluencers with 1k+ followers to Macroinfluencers with 100k+ followers, and even Megainfluencers with 1M+ followers [23]. Influencers interact with followers frequently, through posts and replies to comments, which, in turn, enhances their engagement rate on a social media platform [24,25]. While many influencers are laypersons (ie, without formal training), some health professionals can also become influencers by actively promoting content and building a social media presence (eg, Dr Pal, Dr Mike) [26-28].

The prior studies also demonstrate the role influencers have in potentially promoting harmful and misleading information and ideals (eg, extreme diets and striving for a certain physical appearance) [29,30]. This includes studies that examined the extent of food and nutrition misinformation on social media, with a focus on a particular health condition, such as eating disorders [31], as well as those studies that explored the broader spectrum of false and misleading dietary claims [32].

Other studies of social media content have characterized how knowledge coconstruction of food literacy occurs on social media and the specific nutritional content of recipes demonstrated in social media posts [16,33]. Characterizing social media content using a food literacy framework can improve our understanding of how these topics are being discussed by a broader population, gaps in this landscape of information, and areas for improving nutrition education. Furthermore, food literacy is socially constructed; thus, examining how food literacy is understood by public audiences through their social media posts can then inform practitioners’ and policymakers’ definitions of food literacy and future programming and policymaking. Diet-related illnesses and poor dietary behaviors impact populations from low socioeconomic status backgrounds and racialized populations most frequently due to a complex set of factors, including food insecurity, cultural and social norms, intergenerational trauma, and low food literacy, among others [34]. However, no studies have examined whether and how issues of cultural foods and affordability of foods and food insecurity are represented in social media content.

The primary objective of this study was to systematically characterize and categorize publicly available food- and nutrition-related Instagram content according to food literacy concepts. Our secondary objective was to explore the extent to which low-cost and affordable recipes and foods were represented, including those that reflect cultural foods and eating practices.

## Methods

### Study Design

To address the study objective, we used a mixed methods approach using qualitative and quantitative content analysis [35]. We selected a mixed methods approach as the combination of qualitative and quantitative evidence complements each other and provides a more comprehensive understanding of our research objectives [35]. Given the complexity of social media content and the exploratory nature of this research, studies by other groups suggest that multiple methods allow combinatory strategies to be used to explore and understand the topics of interest [36]. In the collected data, we were interested in what individuals were posting on social media and how frequently certain topics occurred or did not occur.

### Ethical Considerations

This study was not considered research that would require ethics review board assessment by our local institutional research ethics board based on the “SickKids Research Ethics Board: Research Ethics Handbook” and our strategies for maintaining privacy and confidentiality, collection of public posts, and size of the dataset.

### Conceptual Framework

We used the food literacy framework by Vidgen and Gallegos [15] and Canada’s Food Guide [37] to guide our characterization of food- and nutrition-related Instagram posts. We selected the framework laid out by Vidgen and Gallegos [15] because of its focus on the food skills component of food literacy and its development rooted in experiences of individuals from culturally diverse and low socioeconomic status backgrounds. We combined this framework with the Canadian Food Guide, given our aim to develop an intervention for Canadian youth, and the strengths of the guide in articulating social and societal aspects of food literacy and what types of food to consume. We reviewed each framework and identified key themes and topic areas, grouping similar topics together. The final framework that guided our analysis is outlined in Table 1.

**Table 1:** Food literacy conceptual framework, developed by authors based on the Canadian Food Guide [37] and Vidgen and Gallegos [15].

Topic name			Description
**What to eat**			
	Foods to consume	Consume vegetables, fruit, whole grains, and protein foods regularly. Consume plant-based protein foods more often. Replace foods that contain mostly saturated fat with foods that contain unsaturated fat. Water is the beverage of choice.	
	Limiting processed, prepared, sugar-sweetened, and other foods	Processed or prepared foods and beverages with excess sodium, free sugars, or saturated fats should not be consumed regularly as they undermine healthy eating.	
	Abstain from alcohol	Alcohol is associated with several health risks and contributes significant calories with little to no nutritive value. Thus, alcohol should be avoided.	
**How to eat**			
	Eating in a social way	Join with others to eat in a social way. Sharing meals with others can make healthy eating enjoyable and foster connections with cultures and generations.	
	Promoting and maintaining cultural foods^a^ and traditions	Culturally specific foods and food traditions can improve diet quality.	
	Energy needs	Food choice in part based on energy needs, which are individual and depend on multiple factors, including level of physical activity, age, sex, height, genetics, and others.	
	Fad diets	Some fad diets can be restrictive and pose risks (eg, juicing diets and cabbage soup diet).	
**Impact of food**			
	Environmental impact of food	Food choices have an impact on the environment. Plant-based foods may have a lesser environmental impact compared with animal-based foods. The way food is produced, processed, distributed, and consumed, as well as food loss and food waste, all have environmental impacts.	
	Impact of food on well-being	Understand the impact of food on personal well-being.	
	Food labels	Understand how to read food labels to determine a food’s nutritional value, compare nutritional content of food products, and manage special dietary needs, such as allergies and or low-sodium diets.	
	Food insecurity and security	Healthy eating requires healthy foods to be available and accessible. Certain communities and populations are at higher risk of poor dietary intake due to food insecurity.	
**Cooking skills**			
	Planning and managing food intake	The ability to prioritize money and time for food, plan food intake so that it can be accessed irrespective of changes in circumstance, and make food decisions that balance personal needs with available resources. Adequate food skills may reduce food waste.	
	Selecting foods and determining quality, type, and handling	The ability to access food through different sources and understand the advantages and disadvantages of each, and to determine what is in a food product, where it came from, how to store it, and use it, and judge its quality. Nutritious foods can be fresh, frozen, canned, or dried. Ability to use the senses to assess texture, appearance, taste, and smell of foods to determine quality.	
	Preparing food	The ability to make a good-tasting meal from available ingredients. It includes preparing seasonally and commonly available foods, using available pieces of kitchen equipment, and the skills to adapt cooking methods according to food needs and available ingredients and equipment. This includes the ability to practice basic principles of safe food hygiene and handling. Cultural food practices should be celebrated and kept alive by sharing them across generations and with others.	
	Food skills should be taught, learned, and shared in any setting	Teaching and sharing food skills can happen in schools, daycares, community centers, gardens, community kitchens, during family celebrations, during meal preparation, or community celebrations. Food skills are life skills and should be transferred to children and adolescents.	

^a^The term “cultural foods” here is used to refer to foods from non-Western countries and cultures.

### Social Media Platform and Search Platform

We chose to examine social media content on Instagram for this exploratory study. Instagram has high popularity among adolescents, high use among males and females, and existing use among adolescents to search for health information [38]. Other platforms that we considered were TikTok and YouTube. At the time of this study, TikTok may have had fewer users than Instagram among our target population in Canada [39]. We also considered YouTube due to the high use among young people. However, it had lower use among adolescent populations and a high proportion of male users (70% male), which may have limited the study findings to this specific population of users [38].

We used the data collection platform CrowdTangle to collect publicly available Instagram content (photos, videos, and albums) on food and nutrition. CrowdTangle is Meta’s online platform that allows researchers to search all publicly available Instagram posts made by accounts with more than 50K followers, as well as verified accounts [40,41]. As of August 2024, CrowdTangle is no longer available, but Meta has introduced the Content Library and application programming interface, which offer comparable access and search functionality [42]. We were interested in examining influencers’ content as a recent national survey demonstrated that food content was the most popular genre of influencer content in Canada [43]. CrowdTangle was able to collect the Instagram account associated with each post, however, determining the nature of the source account (eg, individual or organization) and the intention of the post was beyond the scope of this study. At the time of the study, CrowdTangle did not support searching and collection of Instagram reels.

### Search Strategy

Due to the broad nature of food and nutrition posts and the exploratory nature of our research objectives, we initially developed a search strategy aiming to identify posts that provide any type of nutrition-related information or education (eg, searching for posts using specific nutrition and cooking-related terms). However, we found that this approach lacked specificity, often returning posts related to animals or other nonfood or nonnutrition related products. Thus, we opted to use a hashtag-focused approach as other studies have done. Hashtag-based search strategies have good specificity, given that hashtags are used by users with the explicit purpose of being identified as relevant to a specific topic. For example, the hashtag #mealplanning may be in posts by users who hope that their post is reviewed by users who are interested in posts about meal planning.

We iteratively developed our hashtag search strategy through (1) brainstorming candidate hashtags, (2) reviewing Instagram posts for additional relevant hashtags to the topics of nutrition and cooking, as described below, and (3) reviewing our conceptual framework. The hashtags were tested individually to ensure that they (1) returned posts and (2) that the posts were relevant to the topic of nutrition and cooking, based on a review of the 30 most popular posts tagged with each hashtag. In the final search strategy, we added a filter to remove posts that were branded or ads as they generally did not contain content relevant to our topic of nutrition and cooking literacy.

The final search strategy included the following 14 hashtags: #healthyeating, #healthycooking, #healthyfood, #nutritioniskey, #healthyeats, #healthyrecipes, #homecooking, #healthydinners, #healthylunch, #healthybreakfast, #healthymeals, #healthysnacks, #familyrecipes, and #foodislove. The frequent use of hashtags containing the word “healthy” in our final selection reflects how users frame and communicate food-related content on Instagram and aligns with previous research on this topic [44,45].

The date range of the posts was April 1, 2022, to May 20, 2022. Instagram posts were retrieved using this search strategy via CrowdTangle. CrowdTangle retrieves Instagram posts, post image text, post captions, comments, number of likes, number of comments, number of views, Instagram account name, number of followers, sponsorship information, and calculates a score that indicates whether the post is over or underperforming relative to the other posts by that account [40].

### Data Analysis

Our mixed methods approach to data analysis began with identifying and quantifying Instagram posts related to the topics in our conceptual framework and research objectives using Netlytic. Netlytic is a cloud-based research tool that allows researchers to automate the process of summarizing textual data, among other features. We iteratively created dictionaries related to each topic of our conceptual framework, generating terms using the framework itself, brainstorm, a literature review, and thesaurus. We revised, added, and removed terms based on a review of 10 to 30 posts that were identified using the dictionaries. We combined categories where there was significant overlap and added categories based on additional areas of interest that we identified in the analytic process. This process resulted in the addition of categories related to attitudes and perceptions, taste of food, good feelings, and bad feelings, as well as an exercise and physical activity category.

The next step in our analysis involved an exploratory qualitative content analysis of a sample of the posts from each topic category. This approach was used to describe the post content and caption in relation to our conceptual framework. The lead author, who has expertise in food literacy programs, served as the sole reviewer. They reviewed a random selection of posts within each category until a consistent understanding of the content emerged. The preliminary results were reviewed by all authors, and any revisions and clarifications were addressed. Since the number of posts varied greatly across categories (from 23 to over 38,000 posts), up to 100 posts were reviewed per category to reach saturation, at which point additional posts no longer provided new insights or themes. The resulting descriptive summaries for each category are presented in the following section.

## Results

### Overview

Our search strategy identified a total of 100,000 Instagram posts made between April 1, 2022, to May 20, 2022, during the time period for this study. We limited the dataset to 100,000 posts to allow for feasibility for review by our research team.

Table 2 outlines the number of posts per category in our content analysis. The categories covered each of the topics in our conceptual framework. We combined the categories of eating in a social way and food skills should be taught, learned, and shared in any setting, as there was significant overlap between the dictionaries and content. Categories were not mutually exclusive. The most common topics of foods to consume for good health and preparing food were addressed by 38,500 of 100,000 (38.5%) and 36,007 of 100,000 (36.0%) posts, respectively, while the topic of planning and managing food intake was addressed by 33,262 of 100,000 (33.3%) posts. Selecting foods and determining quality, type, and handling were addressed by 25,775 of 100,000 (25.8%) posts. The least common topics of alcohol, environmental impact of food, food labels, and food insecurity and security were addressed by 23 of 100,000 posts. Low cost and affordable foods were addressed by 1680 of 100,000 (1.7%) posts. A total of 85,395 of 100,000 (85.4%) posts were categorized through our text analysis. The remaining posts (14,605 of 100,000, 14.6% posts) did not fit into any of the categories, mostly consisting of images without captions.

**Table 2:** Posts per category (N=100,000).

Topic name		Posts, n (%)	Dictionary terms, n
**What to eat**			
	Foods to consume	38,500 (38.5)	111
	Limiting processed, prepared, sugar-sweetened, and other foods	4511 (4.5)	30
	Abstain from alcohol	128 (0.1)	1
**How to eat**			
	Promoting and maintaining cultural foods and traditions	14,865 (14.9)	281
	Energy needs	20,135 (20.1)	17
	Popular/Fad diets	11,299 (11.3)	19
**Impact of food**			
	Environmental impact of food	118 (0.1)	4
	Impact of food on well-being	17,247 (17.2)	20
	Food labels	83 (0.1)	4
	Food insecurity and security	23 (0.0)	5
**Cooking skills**			
	Planning and managing food intake	33,262 (33.3)	27
	Selecting foods and determining quality, type, and handling	25,775 (25.8)	24
	Preparing food	36,007 (36.0)	45
	Eating socially and food skills should be taught, learned, and shared	11,386 (11.4)	16
**Additional topics**			
	Low-cost and affordable foods and recipes	1680 (1.7)	21
	Exercise and physical activity	16,152 (16.2)	7

### Content Analysis of Categories

#### Foods to Consume

Posts in this category included images of foods containing protein, such as chicken, eggs, tofu, and protein powders, and terms related to protein-containing foods. Posts provided recipes for high-protein meals and smoothies. Vegetable-related images and terms were the second most common and largely described as “greens” and images largely included vegetables that were green in color. These posts also included images and captions about green juices and green tea, describing the healthfulness and benefits for weight loss and “detox” for smoothies, juices, and green tea. In this category, nutrition claims about foods focused on fiber and cholesterol, and meeting protein intake requirements. Spices and water-related terms were the third and fourth most common in this category. Spice-related terms included images and captions related to cultural foods, holidays, and cultural practices.

#### Limiting Processed, Prepared, Sugar-Sweetened, and Other Foods

Posts in this category encouraged individuals to avoid preservatives and processed foods. None of the sampled posts described alternatives to foods that are prepared, processed, or sugar sweetened. Nor did posts provide further information regarding preservatives in foods.

#### Abstain From Alcohol

All posts in this category encouraged avoiding and abstaining from alcohol. Posts described the health impacts of avoiding alcohol, including those associated with the calories from alcoholic beverages.

#### Promoting and Maintaining Cultural Foods and Traditions

This category included many posts by non-Western cuisine restaurants and businesses. Multicultural recipes were portrayed as healthy. They included ingredients such as whole grain wheat (eg, buckwheat dosa), healthier variations of classic recipes (eg, low-carbohydrate Filipino desserts), and recipes for specific conditions and people (eg, Indian postpartum foods).

#### Energy Needs

Posts covering energy needs are often related to fitness and workout-related terms and images. Consuming healthy food, especially high-protein foods, were described as a method of improving fitness and workouts.

#### Popular/Fad Diets

Posts in this category frequently portrayed dietary habits such as keto diets, paleo diets, and smoothie or juicing-only diets as necessary for healthy living and weight loss. These diets were portrayed as healthy and as healthier options for the general population and as options for weight loss. Other posts portrayed different dietary practices such as “cleansing,” diet challenges where processed and sugar added foods are avoided completely, and the benefits of organic foods.

#### Environmental Impact of Food

This category of posts focuses on food waste most frequently, followed by the carbon footprint of food products. Selecting plant-based foods was portrayed as a method of reducing carbon footprints. Strategies to reduce food waste to save money and avoid environmental impacts were portrayed, including how to use food of varying stages of freshness, and how to keep food fresh for longer. Many posts were also related to Earth Day, which was captured in the period of our sample (April 22, 2021).

#### Impact of Food on Activities and Well-Being

The impact of food on workouts was the most frequent topic in this category, followed by the impact of food on mood (eg, feeling happy) and energy levels. Consuming healthy foods was portrayed to improve feelings of sluggishness and energy levels. Unlike the Energy Needs category, which emphasizes what the body requires to support physical activity, this category focuses on how food is portrayed as influencing overall well-being.

#### Food Labels

Posts in this category provided general advice about reading food labels as well as tips for selecting foods based on their ingredient lists. Common tips for reading ingredient lists included foods with shorter ingredient lists are considered healthier, and avoiding ingredients that are difficult to pronounce. Businesses would promote their product’s nutrition fact label as a sign of healthfulness. Posts also addressed how to read labels to identify dietary restrictions and vegan or vegetarian products. These posts also included the nutrition facts associated with recipes provided in the post.

#### Food Security and Insecurity

Posts in this category highlighted efforts to address food insecurity through community initiatives and partnerships. These included programs that distributed groceries to households or provided budgets for families to purchase fresh foods and groceries. Other posts promoted products that were positioned as options for improving food security, particularly in the context of climate change affecting food security. Some content emphasized the broader role of climate in food security.

#### Planning and Managing Food Intake

Posts in this category focus on easy and quick recipes in terms of preparation and purchasing. Planning food intake was often in relation to diets and weight management plans with the aim of weight loss. Planning food intake was also frequently discussed in relation to activities and holidays, especially those occurring during the same time period as the data collection window. For example, Ramadan (iftar, fasting), Navratri (fasting). Posts in this category often included content by vendors offering their meal plan services.

#### Selecting Foods and Determining Quality, Type, and Handling

Posts in this category covered differences between fresh, frozen, and organic foods and preferences for one over the other. Fresh foods were the preferred options for many recipes, while frozen foods, mainly fruits and vegetables, were portrayed in relation to smoothies and homemade juices. Freezing was also a common method portrayed for storing herbs and preventing food waste. Restaurant foods were portrayed as options to be selected for socializing and for holidays (eg, Ramadan and Ugadi).

#### Preparing Food

In this category, baking-related terms were most frequent. The pan was the most frequently mentioned cooking appliance. Cooking more often included posts and recipes from non-Western accounts, whereas baking more often included posts and recipes from Western accounts. Posts in this category included recipes in the post itself or linked to recipes elsewhere. Terms and posts related to ingredients would describe choosing and selecting ingredients as well.

#### Eating Socially and Food Skills Should Be Taught, Learned, and Shared

Posts in this category focus on preparing food in a social way more frequently than simply eating together in a social way. Sharing recipes with friends and family members was common and used as an engagement strategy, asking users to share recipes with peers. Cooking together was commonly depicted as an enjoyable activity.

#### Low-Cost and Affordable Foods and Recipes

Posts in this category portrayed budget-friendly or simple recipes, using minimal ingredients, minimal time, and affordable vegetables. Posts also portrayed strategies such as using sales to maintain a budget. Many posts portrayed recipes and strategies as “family-friendly.” Posts in this category also included many from manufacturers advertising meal and cookware products as affordable.

## Discussion

### Principal Findings

In this exploratory study, we characterized and categorized a sample of healthy eating related Instagram posts according to food literacy concepts. In our sample, hashtags were most frequently related to Instagram communities focused on healthy living and food photography. In our analysis, we categorized Instagram posts using 19 categories related to food literacy. We found the largest categories to be the “foods to consume” category, “preparing food,” and “planning and managing food intake.” These categories of information reflect important components of food literacy. Posts promoted protein-rich foods, vegetables, high fiber foods, low cholesterol foods, juicing and smoothie diets, and spices as foods to consume, and encouraged selecting organic and fresh foods over canned or frozen foods. Posts frequently portrayed baking as a cooking method and quick and easy recipes. Planning food intake was depicted in relation to weight loss and holidays. Food was portrayed as having an impact on fitness, energy levels, and mood. Cultural foods were depicted as healthy foods and with healthier variations, as well as in the context of holidays and religious observances. Cooking together and sharing recipes with friends and family were more frequently portrayed than simply consuming meals together. Restrictive diets were frequently portrayed as healthy and as the alternative or opposite to consuming processed, prepared, and sugar-sweetened foods, all of which users were encouraged to avoid (see Results: “Popular/Fad Diets” subsection). Low-cost and affordable foods were portrayed with minimal time requirements, minimal ingredients, and depicted as family-appropriate. All posts related to alcohol encouraged avoiding and abstaining from alcohol consumption. Posts related to the environmental impact of food focused on reducing food waste and the carbon footprint of groceries. Posts covering food labels provided general advice to read food labels, however lacked specific guidelines for reading food labels. Posts covering food insecurity aimed to promote awareness of food insecurity in the community and highlight initiatives aiming to reduce food insecurity. These findings demonstrate social media users’ and content creators’ interest in nearly all aspects of food literacy as well as several opportunities for improving food literacy education on social media.

### Most Frequent Terms and Hashtags

The most used terms and hashtags in our sample related to healthy living and food-related Instagram communities. Instagram users’ post descriptions discussed “healthy eating” with healthy living and healthy lifestyles. Prior studies have also identified similar patterns in analyses of the “healthy food” hashtag on Instagram and on Twitter, where healthy eating is portrayed as a component of a healthy lifestyle [45,46]. Among food photography Instagram communities, healthy eating was frequently accompanied by terms describing good-tasting food and images of appetizing food. Thus, there appears to be a tendency for healthy foods to be the focus of food photography communities, suggesting that healthy eating is seen as visually appealing or good-tasting. In our sample, we noticed that diet and weight loss hashtags occurred less frequently and as a smaller proportion of the posts. This contrasts with a prior study of the “healthy food” hashtag on Instagram conducted in 2021 [46]. The study authors found that weight loss was frequently associated with the term [46]. Part of this discrepancy in findings may be due to differences in analytic strategy. The authors of that study grouped exercise- and fitness-related terms with diet and weight loss terms, whereas our study separated the terms. Our interpretation of these findings is twofold, first, weight loss as a concept may not be as frequently associated with healthy eating as it is with exercise as a method of weight loss. A study of #fitspiration posts—a hashtag associated with images of healthy approaches to weight loss and fitness—on Instagram, Facebook, Tumblr, and Twitter (renamed X in 2023) found that posts were more frequently related to exercise (70% of posts) rather than food and eating (16% of posts) [47]. Second, communities and individuals on Instagram may have currently trended away from the weight loss aspects of healthy eating. There may be a growing recognition among users of the impact of healthy eating on health and well-being, rather than simply a means of weight loss [48,49].

### Content Analysis of Instagram Posts

Instagram content depicted high-protein foods as an important food to consume, likely due to fitness- and exercise-related content promoting high-protein foods and protein supplements. A qualitative study in 2017 with users who consumed fitness and exercise content by influencers who also promoted protein supplements suggested that the portrayal of these foods is mainly tied to commercial influence and partnerships with influencers [50].

Vegetables, especially green vegetables, were frequently portrayed as foods to consume, likely due to their relation to “fitspiration” communities and mirroring existing influencers and content creators promoting healthy eating [51]. Holmberg et al [51] conducted an exploratory study of adolescents’ food-related Instagram posts and found that depictions of fruits and vegetables frequently resembled influencer food content and cookbook food images. Smoothies, juicing, green tea, and detox diet food were also portrayed as foods to consume, which may be due to sustained misinformation as well as the view that smoothies and juicing are effective ways of increasing fruit and vegetable intake. In terms of nutrients, fiber and cholesterol were frequent topics, where increasing fiber was encouraged due to its role in improving bowel movements and gut health, and reducing cholesterol to improve cardiovascular health.

Encouragement to cook with spices appeared to be tied to preparing cultural foods rather than general cooking practice. This is likely due to a more limited view of spices as a part of non-Western foods and cultures. One study of spice and herb use among US adults found that spice and herb use and self-efficacy were significantly higher among non-White adults and suggested that this was due to a higher use of spices in foods prepared in regions such as India, Mexico, and Vietnam [52]. Furthermore, there may be a misunderstanding of spices and their role in cooking. Spices can have health benefits and increase the palatability of vegetables; however, studies of adults’ and adolescents’ perceptions of spices in the United States suggest limited knowledge and understanding of these benefits [53,54].

In terms of preserved foods, we found few depictions of canned vegetables and dried foods (eg, beans and lentils). This likely stems from negative perceptions of canned foods and dried foods in general either as unsavory or unhealthy, as well as less aesthetically pleasing in Instagram content. A study of US parents’ perceptions of different food types found that both immigrant and US-born parents viewed canned foods as processed and unhealthy [55]. A study with science teachers in Jordan also found a widespread belief that canned foods were unhealthy due to processing and high carbohydrate content [56]. These perceptions may be pervasive in a greater proportion of the population and, as such, are reflected in limited Instagram content depicting canned and dried foods. Canned vegetables and beans can often be affordable, convenient options for vegetables and protein, especially for low socioeconomic status populations [57]. Furthermore, canned food consumption may be associated with greater vegetable, protein, and fiber intake [57].

Posts related to alcohol shared information in line with Canadian alcohol consumption guidelines, which recommend abstaining or limiting alcohol consumption to 2 drinks per week to avoid and limit the risks associated with alcohol consumption, respectively [58]. However, the lack of posts addressing the link between alcohol and negative health outcomes may prevent users from understanding why current guidelines suggest abstaining from alcohol or limiting alcohol intake may benefit their health. Since none of the 14 hashtags used targeted alcohol or beverage-related keywords, future studies should include alcohol-focused hashtags to better capture this aspect of food and nutrition content on Instagram.

We found that while recipes were frequently depicted in Instagram content, few posts focused on specific cooking skills or methods of cooking or recipes that could translate to different ingredients, amounts of time, and equipment. This is likely because such a level of granularity in teaching cooking is more difficult to depict in a way that generates high engagement rates. Furthermore, it is likely that when social media users turn to Instagram for cooking and food content, they are looking for recipes that can be followed step by step and require little thought with respect to substitution or modification. Social media users may also believe they do not require instruction in cooking skills that they view as more basic, such as knife skills when chopping vegetables, how to prepare meat, or how to use a can opener. Food preparation skills, as outlined in our conceptual framework, suggest that individuals should be able to use ingredients they have available to them to prepare a meal using cooking methods with which they are comfortable and suited to the meal they are preparing. While recipes can introduce users to new foods, flavors, or methods of preparation, they can also limit users with the assumption that cooking as a process should be followed in a step-by-step manner. Exploring Instagram reel content and TikTok content may allow us to explore this issue further to identify whether video-based content is better able to demonstrate cooking as a skillset rather than the rigid use of step-by-step recipes. In terms of methods of preparing food, posts depicting baking and baking desserts were most common, similar to other studies, which have found dessert-based recipes and baking recipes as the most popularly portrayed. This is likely due to the strong interest and popularity of baking as a hobby for many individuals. Quick and easy meal preparation methods were also most frequently portrayed, likely due to social media users looking for meals that allow greater convenience, as well as avoiding the impression for users that recipes are outside their skill level. The promotion of meal planning and delivery services suggests a rise in these services to meet the demand for convenience in preparing and consuming healthier meals.

A few Instagram posts shared strategies for food label reading. The strategies that were shared were simple (eg, avoid foods with ingredients that you are unable to pronounce) and did not address nutrients listed on food labels (eg, fiber and protein). Food labels tend to be one of the more overwhelming topics in food and nutrition education; yet, in this dataset, we found limited content addressing this topic [59-61].

The impact of healthy eating on health and well-being focused on energy and exercise, rather than longer-term impacts such as nutrient deficiencies, preventing cardiovascular disease, and other issues contributing to chronic disease development. This may be because eliciting dietary changes with impacts that may be seen immediately are easier to communicate and convince social media users of, rather than impacts that are longer-term and difficult to imagine or understand. Posts in the category of Popular/Fad Diets presented dietary practices such as juicing or paleo diets as suitable for all individuals. However, the paleo diet, for instance, is not suitable for those at higher risk of cardiovascular disease or with increased calcium needs. These posts reflect historical trends in special diets for weight loss and the perception that healthy eating is a means for weight loss alone rather than a means for a healthy lifestyle. Addressing this misinformation and providing accurate nutrition information may be an important approach in nutrition promotion.

In our dataset, cultural foods were portrayed as healthy foods and as foods that could be made healthier with different ingredients. This impression may be driven by increased awareness that cultural foods from restaurants or that are purchased prepared may be more detrimental to health, and by many non-North American users posting typical foods for their country. As in the past and present, many cultural diets have been unfairly portrayed as unhealthy or detrimental to health, causing negative perceptions around cultural foods and stigma towards certain diets [62]. We were unable to find other literature reports of social media content covering the planning of food intake around religious and cultural observances, which require special diets or fasting. Our dataset covered the period of Ramadan and Navratri, which may explain the number of posts relating to fasting or holidays. These findings suggest that social media users are likely looking for guidance to maintain good health through their diet during fasting or temporary dietary restrictions.

In our analysis, we combined the eating in a social way category dictionary with the food skills should be taught, learned, and shared in any setting category as we discovered Instagram posts focused on preparing and cooking food in a social way rather than simply eating meals together. Eating meals socially was depicted more frequently with cooking rather than as a standalone depiction. In the content we analyzed, cooking was perceived as a social activity with recipes being a common way of sharing cooking interests asynchronously. Our initial framework covered these 2 concepts separately; however, guidance on healthy eating and food literacy may need to be revised to reflect the relationship between cooking together and sharing meals together, and the importance of cooking as a social and enjoyable activity.

Posts in the category of low-cost and budget-friendly foods depicted these foods as simple and easy, requiring limited ingredients and time. This perception of low-cost foods, while enticing for individuals looking for ways to simplify cooking and meal planning, can cause the perception that budget-friendly foods are necessarily easy, less interesting, and less flavorful. These ideas can cause individuals to move away from low-cost recipes or ingredients, which they may perceive to be less tasty and interesting to prepare. Cooking (aside from its cleanup), as evidenced by the number of food communities on Instagram, is viewed as a fun activity for many people, and attaching a perception that lower-cost meals are less valuable or enjoyable can distort their appreciation and involvement in it.

Posts regarding the environmental impact of food focused on food waste (eg, household food waste) more frequently than impacts of food production on the environment (eg, dairy and beef farming). Posts alluded to plant-based foods having a smaller environmental impact; yet, this was more frequently portrayed when comparing animal dairy products to plant dairy products or plant-based meat alternatives. The environmental impact of plant-based dairy products and meat alternatives is variable when compared with animal-based products [63]. Furthermore, plant-based dairy and meat alternative products are costlier, especially when feeding families and larger groups of people [64]. This issue can cause youth and other individuals to believe that their purchasing of these products is directly detrimental to the environment and, as a result, become stigmatizing [65].

In this dataset, we identified a few posts addressing food security or insecurity. This finding contrasts with the growing issue of rising food insecurity in many countries [66]. The lack of discussion around food insecurity may be due to the terms and phrases used in our dictionary for the category of food security. In academic and policy settings, terms such as food security and insecurity are generally used; however, social media users may use different terms and phrases to describe these issues [67]. Another possibility is that food security and insecurity are not significant topics of discussion on social media [68,69]. If this is the case, further investigation may be required to understand the reasons for this trend. A component of food literacy is the understanding and appreciation of food security as part of the societal-level factors influencing one’s ability to select, choose, and prepare foods that promote health [15]. Interestingly, posts that did address food insecurity discussed the role of climate change in food insecurity, rather than other factors such as the role of governments and industry.

### Strengths and Limitations

There are several limitations to this exploratory study. First, there were limitations due to the data collection platform used; at the time of the study, CrowdTangle did not collect reels or videos from Instagram. Thus, this work is limited to characterizing photo and image-based content. Given that a shift towards video and reel-based content was occurring at the same time as this study, we hypothesize that the data collected and study results may be generalizable to video and reel content on Instagram at that time. Second, the search strategy in this study used a hashtag-based approach to collect posts to ensure a broad collection of posts. The limitation of this approach is that some Instagram users and accounts do not use hashtags in their posts, limiting the content that was collected from users who do not use hashtags, who may potentially differ from users who do use hashtags. We were unable to identify literature characterizing the specific differences between users who use hashtags and those who do not. Finally, our investigation of cultural foods among the posts was limited by the dictionary we created, which focused on country names and demonyms.

The strengths of this study are multifold. First, we used a framework of food literacy informed by the work of Vidgen and Gallegos [15] and Canada’s Food Guide [37] to guide this study from design to analysis. The combined framework we created for this study allowed us to achieve appropriate depth and breadth in characterizing the food- and nutrition-related content on Instagram in this exploratory study. Second, the constant comparison method we used in our analysis to categorize content that fit within the framework and content that required an expansion of the framework allowed us to suggest implications and refinements to current education and policy. Third, we used an iterative approach in the design and validation of both our search strategy and analysis to ensure our dataset was relevant to the topic of healthy eating as well as the experience of healthy eating as shared by Instagram users.

### Conclusions

This is the first study to characterize Instagram content on healthy eating. Our findings demonstrate how healthy eating is currently conveyed on social media. Healthy eating is more frequently portrayed as part of a healthy lifestyle, impacting activity, energy levels, and mood. However, content representing restrictive diets and approaches to eating still pervades the user generated content. Low-cost and affordable foods and cooking are more frequently represented as simple and easy, which may impact public perceptions of the taste of affordable foods and meals. Few posts addressed food label reading accurately or adequately, representing a potential opportunity for future public health campaigns. Finally, social media users depicted the importance of cooking and eating together and cooking as a social and enjoyable activity. Future iterations of the guide may revise the guideline to reflect how individuals currently approach cooking together more importantly than eating together. The lack of posts regarding food insecurity suggests a need to strengthen this aspect of food literacy education.

## Data Availability

The datasets generated or analyzed during this study are available from the corresponding author on reasonable request.
